# Prognostic Models for Incident Hypertension: Time to Move from Development to Implementation—An Umbrella Review

**DOI:** 10.3390/diagnostics16193069

**Published:** 2026-09-22

**Authors:** Víctor Juan Vera-Ponce, Jhosmer Ballena-Caicedo

**Affiliations:** Facultad de Medicina (FAMED), Universidad Nacional Toribio Rodríguez de Mendoza de Amazonas (UNTRM), Chachapoyas 01001, Peru; 7330178022@untrm.edu.pe

**Keywords:** hypertension, prognostic models, risk prediction, machine learning, external validation, umbrella review, implementation gap, clinical utility

## Abstract

Hypertension represents a major modifiable cardiovascular risk factor with increasing global prevalence. While numerous prognostic models for incident hypertension have been developed, their clinical implementation remains limited, suggesting a gap between model development and practical application. This study aimed to synthesize evidence from systematic reviews on multivariable prognostic models estimating the future risk of incident hypertension among initially normotensive individuals, evaluating methodological quality, predictive performance, validation status, calibration reporting, and clinical utility. We conducted an umbrella review, searching six databases from January 2000 to March 2026. Systematic reviews (SRs) evaluating multivariable prognostic models for incident hypertension with documented external validation evidence were included; reviews mixing prognostic and cross-sectional diagnostic screening models were retained only when eligible prognostic evidence could be distinguished and were examined in sensitivity synthesis. Methodological quality was assessed using ROBIS, and primary-study risk of bias was extracted from PROBAST/CHARMS assessments reported by the included reviews. Six SRs (three with meta-analyses) identified 162 unique models reported as prognostic. Models were categorized as traditional statistical (60%), machine learning (30%), and genetic/polygenic risk score-based (10%). Reported pooled AUCs were 0.78 (95% CI 0.76–0.80) for traditional models and 0.82 (95% CI 0.77–0.86) for machine learning models, but all quantitative summaries showed extreme heterogeneity (I^2^ > 99%) and should therefore be interpreted descriptively rather than as stable estimates of clinical performance. Only 21 of 94 models with extractable validation information (22%) underwent at least one external validation, and only seven of 162 unique models were externally validated more than once. Calibration was reported in a minority of models, and no published clinical-impact studies evaluating the use of these models in routine practice were identified. The field should avoid prioritizing additional de novo model derivation in already well-represented settings and should redirect resources toward external validation, recalibration, clinical-impact evaluation, and implementation of the most robust existing models in primary care.

## 1. Introduction

Hypertension (HTN) represents one of the principal modifiable risk factors for cardiovascular disease (CVD), substantially contributing to the global burden of morbidity and mortality. With a worldwide prevalence reaching 30–45% of the adult population and an increasing trend, especially in low- and middle-income countries, HTN constitutes a priority challenge for public health [1]. The frequently asymptomatic nature of this condition until advanced stages underscores the importance of preventive strategies and early detection based on identifying individuals with a higher risk of developing it.

In recent years, multivariable prognostic models have integrated several risk factors into a single probabilistic estimate for different pathologies, including HTN, facilitating population stratification and clinical decision-making to prevent this disease. During the last decades, there has been an exponential growth in the development of these models, evolving from traditional statistical approaches such as logistic regression to incorporating advanced machine learning (ML) techniques and genetic biomarkers [2]. However, this rapid development has raised concerns about these models’ methodological quality, external validation, and clinical applicability, critical aspects of their effective implementation.

The proliferation of prognostic models for HTN has been accompanied by several systematic reviews (SRs) attempting to synthesize the available evidence. These studies have evaluated aspects such as the development methods, performance, validation, and clinical implementation of the models, generating a body of secondary literature that requires a higher-level synthesis [3,4]. Nevertheless, their methodological approach, inclusion criteria, and search periods differ, generating discrepant conclusions and hindering interpretation for researchers and clinical professionals.

Primary care settings represent the cornerstone of hypertension detection and management globally, with studies indicating that 70–90% of hypertension diagnoses and initial management occur at this level of care [5]. A systematic analysis of healthcare utilization patterns demonstrates that primary care physicians manage approximately 85% of all patients with hypertension, serving as the first point of contact for screening, diagnosis, and long-term management [6]. This central role is particularly critical in resource-limited settings, where primary care may be the only accessible healthcare level for most of the population [7]. Despite this fundamental role, the development and validation of prognostic models for hypertension have predominantly occurred in specialized research centers and tertiary care settings, creating a significant disconnect between where these tools are created and where they would provide the greatest clinical impact [8].

The present umbrella review (UR) aims to synthesize the available evidence from SRs on prognostic models for incident HTN, critically evaluating their methodological quality, the methods employed by the models, their predictive performance, external validation, and potential clinical applicability. This synthesis seeks to provide a comprehensive picture of the current state of knowledge, identify persistent gaps, and suggest directions for future research, with the ultimate goal of facilitating the translation of these models into clinical practice and contributing to the reduction in the global burden of hypertension.

## 2. Materials and Methods

### 2.1. Research Design

This umbrella review was conducted and reported in accordance with the PRIOR statement [9]. PRISMA 2020 [10] informed the search and study-selection procedures, and methodological guidance for umbrella reviews and pre-diction-model appraisal was also considered [11,12,13]. The completed PRIOR checklist is provided as Supplementary Material S1, and the database-specific search strategies are provided as Supplementary Material S2.

### 2.2. Eligibility Criteria

SRs with or without meta-analyses that evaluated multivariable prognostic models designed to predict the future incidence of HTN in an initially normotensive adult population were included. To ensure a minimum of evidence on model generalization, included reviews had to evaluate at least one prognostic model with documented external validation. No restrictions were imposed based on publication language. Searches were initiated from 2000, considering that research on clinical prediction models underwent significant methodological development from that period, with the publication of specific evaluative frameworks.

For this review, a systematic review was defined as a review that used explicit eligibility criteria, a reproducible search strategy, structured study selection, and a systematic synthesis of included evidence. Reviews without explicit systematic methodology were considered narrative reviews and were excluded.

For the purposes of this umbrella review, prognostic models were defined as multivariable tools estimating the probability of future incident hypertension among individuals who were normotensive at baseline over a stated or inferable prediction horizon. Diagnostic or screening models were defined as tools intended to identify current or previously undiagnosed hypertension at the time of assessment, irrespective of future disease incidence. Reviews including both diagnostic/screening and prognostic models were retained only when they included eligible prognostic models for incident hypertension; diagnostic/screening models were not counted as eligible prognostic models. Borderline cases were adjudicated independently by both reviewers using three criteria: baseline normotensive status, temporal separation between predictors and incident hypertension, and the stated intended use of the model.

Studies that met any of the following criteria were excluded: (1) exclusive focus on models for cross-sectional screening or current diagnosis of hypertension without extractable prognostic models for incident hypertension; (2) evaluation only of models predicting complications or prognosis among patients already diagnosed with hypertension; (3) purely methodological analyses without evaluation of predictive performance in specific populations; (4) narrative reviews, editorials, or commentaries without explicit systematic methodology; (5) reviews that exclusively included models without any external validation; or (6) duplicate publications or preliminary updates of already included SRs.

For the operational definition of incident HTN, we adopted that established by each included review. Most reviews employed the criteria defined by international guidelines contemporary to their publication, mainly the standards of the Joint National Committee (JNC-VII) and the European Society of Hypertension/European Society of Cardiology (ESH/ESC): systolic blood pressure ≥ 140 mmHg and/or diastolic blood pressure ≥ 90 mmHg, or initiation of antihypertensive pharmacological treatment [14]. It should be noted that some more recent SRs (published after 2017) also considered the updated definition of the American Heart Association/American College of Cardiology (AHA/ACC) that establishes lower thresholds (≥130/80 mmHg), although this did not constitute an independent eligibility criterion [15].

### 2.3. Information Sources and Search Strategy

Systematic bibliographic searches were performed in six main electronic databases: MEDLINE (via PubMed), Embase, Scopus, Web of Science (all collections), LILACS, and Epistemonikos. Without language restrictions, the time window spanned from January 2000 to March 2026. The starting point of 2000 was chosen because research on clinical prediction models underwent significant methodological development from this period, with the publication of foundational evaluative frameworks for model development and validation [16,17]. Additionally, our comprehensive searches identified no systematic reviews of hypertension prediction models published before this date. The search strategies were developed by the research team, with final evaluation by a librarian specialized in biomedical information, following a tripartite conceptual structure that combined controlled vocabulary terms (MeSH, Emtree) and free-text terms in title, abstract, and keywords.

The three conceptual blocks of the strategy were: (1) HTN, (2) prediction or prognostic models, and (3) SR. For this last block, validated methodological filters were implemented and adapted according to the characteristics of each database.

The process for developing the search strategies began with PubMed. From there, the strategy for the other databases followed a similar conceptual structure, adapting the syntax and specific controlled terms for each platform. The complete plan, including all Boolean operators, truncations, search fields, and limiters, was fully documented and available in Supplementary Material S2.

### 2.4. Study-Selection Procedure

To complement the electronic search and minimize the risk of omissions, two additional strategies were implemented: (1) manual review of the reference lists of all included SRs (retrospective “snowball” technique); and (2) consultation of prospective SR records, mainly PROSPERO (International Prospective Register of Systematic Reviews), to identify recent or ongoing SRs.

The results of the bibliographic searches were imported into Rayyan QCRI for automated deduplication with manual verification, optimizing the process’s efficiency and minimizing omissions. In the first phase, the two reviewers (VJVP and JBC) independently evaluated the titles and abstracts of all identified records, using the blinding function to prevent mutual influence biases. Articles classified as potentially eligible advanced to full-text evaluation, where both reviewers independently applied a standardized form that systematically verified each inclusion and exclusion criterion.

Disagreements were resolved primarily through direct discussion between the two reviewers; unresolved disagreements, if present, were planned for adjudication by consensus after rechecking the full text and extraction record.

### 2.5. Data Extraction

A standardized data extraction template was developed in REDCap (Research Electronic Data Capture) format, structured into six modules: basic bibliographic characteristics, methodology of the SRs, attributes of the prognostic models, measures of predictive performance, validation methods, and conclusions and recommendations.

Before its implementation, the template was piloted with three SRs with different methodological characteristics, which allowed for refining the extraction fields, clarifying operational definitions, and optimizing the structure to adequately capture the heterogeneity among these studies. This piloting was essential to adapt the template to the particularities of the various types of prognostic models (traditional statistical, ML, and genetic).

The two reviewers (VJVP and JBC) independently extracted data and received specific training in interpreting predictive performance measures and model validation methods. For articles with complex statistical metrics or specialized ML terminology, a biostatistics expert was consulted to ensure interpretive accuracy.

Similarly to the previous section, identified discrepancies were resolved through direct consultation of the original articles in joint discussion, documenting the final decisions in a conflict resolution record that ensured consistency throughout the process.

### 2.6. Methodological Quality and Risk of Bias Assessment

The risk of bias in the included systematic reviews was assessed using the ROBIS tool (Risk of Bias in Systematic Reviews) [18]. ROBIS is specifically designed to assess the risk of bias in systematic reviews, evaluating four key domains: study eligibility criteria, identification and selection of studies, data collection and study appraisal, and synthesis and findings. Each domain contains multiple signaling questions that are answered as “Yes,” “Probably Yes,” “Probably No,” “No,” or “No Information,” leading to a domain-level judgment of “Low Risk,” “High Risk,” or “Unclear Risk” of bias. The overall risk of bias is then determined based on the assessments across all domains and consideration of whether the review methods are likely to minimize bias.

This structured approach allows for a comprehensive evaluation of the review methodology, identifying potential limitations in search strategies, study selection processes, data extraction methods, quality assessment of primary studies, and synthesis of findings. The final classification categorizes each review as having “Low,” “High,” or “Unclear” overall risk of bias, providing a clear indication of the reliability of the review’s conclusions.

Additionally, to obtain a comprehensive view of the risk of bias in the primary studies, we extracted and synthesized PROBAST-AI assessments [13], when used, and PROBAST assessments [19] reported in the included reviews. This ‘review of evaluations’ approach allowed us to aggregate data on the proportion of primary studies classified as low, moderate, or high risk of bias in each of PROBAST’s four key domains: participant selection, measurement of predictors, outcome assessment, and statistical analysis.

The two reviewers (VJVP and JBC) performed all evaluations independently, and any disagreements were resolved through discussion.

### 2.7. Evidence Overlap

A study-review matrix was constructed to assess the degree of overlap between the included SRs, indicating which primary studies were included in each systematic review. From this matrix, the Corrected Covered Area (CCA) [20] was calculated, following the formula:

CCA = (N − r)/(rc − r)

where

- N is the total number of inclusions of primary studies (counting repetitions).
- r is the number of unique primary studies included in all reviews.
- c is the number of included SRs.

The interpretation of CCA followed the established criteria: 0–5% represents slight overlap, 6–10% moderate, 11–15% high, and >15% very high.

### 2.8. Results Synthesis

The findings were synthesised through a structured narrative approach of the six included SRs. First, the general characteristics of these studies were analyzed, followed by an evaluation of their methodological quality and the degree of overlap between them to quantify this redundancy and implement strategies that mitigate its impact on interpretation.

For the identified prognostic models, the synthesis followed a systematic process: categorization according to methodological approaches, analysis of predictor variables used, evaluation of predictive performance in its multiple dimensions (discrimination, calibration, reclassification, and predictive values), extent of external validation, and evidence of clinical impact. For predictive performance, the values of the area under the ROC curve (AUC) reported by each review with their confidence intervals, heterogeneity measures, and prediction intervals were extracted when available, maintaining the original integrity of these results without performing new quantitative syntheses that could introduce additional biases.

Data were also extracted on the percentage of models reporting calibration measures, the proportion that had undergone external validation, the range of positive and negative predictive values, and the presence of reclassification metrics mentioned earlier. The number of external validations per model and their results regarding discrimination and calibration were especially documented. Additionally, the existence or absence of studies evaluating the clinical impact of these models in healthcare practice was systematically recorded.

Finally, information on the methodological quality of the primary studies was synthesized to identify consistent patterns of strengths and deficiencies across the reviews. The percentages of studies with high, moderate/unclear, and low risk of bias in each PROBAST domain (participants, predictors, outcomes, and analysis) were extracted when reported by the included reviews.

Because Sun (2017) [21] and Chowdhury [2] included cross-sectional diagnostic screening models together with true prognostic models, we conducted a restricted prognostic sensitivity synthesis excluding these two reviews from the primary interpretation of prognostic discrimination. These reviews were retained for descriptive mapping of the broader literature but were not treated as primary evidence for the clinical transportability of prognostic models for incident hypertension.

Assessment of small-study effects and publication bias at the umbrella-review level relied on what was reported by the included reviews. We did not construct new funnel plots or conduct Egger-type tests because the relevant quantitative syntheses pooled heterogeneous models and overlapping primary studies, making the unit of analysis unsuitable for a valid overview-level publication-bias test.

## 3. Results

### 3.1. Study Selection

The initial search identified 19,727 records through databases: Scopus (7122), Embase (3919), PubMed (1900), Web of Science (3529), Epistemonikos (2480), and LILACS (777). After eliminating duplicates, 14,844 records were obtained, which were subjected to screening. Fourteen thousand eight hundred twenty-six records were excluded mainly for not being systematic reviews or meta-analyses of hypertension (*n* = 10,088), not including predictive models (*n* = 4256), and being initially undetected duplicates (*n* = 482). Eighteen articles were evaluated in full text, with 10 being excluded for presenting models not specific to HTN (*n* = 10) and not being specifically systematic reviews (*n* = 2). Finally, six studies were included in the narrative synthesis [2,3,4,21,22,23]. Figure 1 presents the PRISMA flow diagram detailing the selection process.

### 3.2. Characteristics of Included Systematic Reviews

The six reviews, published between 2013 and 2024, covered search strategies from 1990/1992 to December 2022. Together, they identified 215 primary studies (counting overlaps), from which 162 unique prognostic models for HTN were described (see Table 1).

The oldest reviews—Echouffo-Tcheugui [22] and Sun [21]—concentrated on traditional statistical models. From 2022 onwards, SRs began to incorporate ML algorithms systematically: Chowdhury [2] and Silva [23] directly compared classical regression with ML approaches, showing similar performances but high heterogeneity, while Schjerven (2024) [4] updated the landscape with separate meta-analyses for both methods. For its part, Hamoen (2019) [3] focused on pediatric models that predict adult HTN from childhood data.

Most models were developed in North American, European, and Asian cohorts, with little or no representation from Africa and Oceania. In all studies, the definition of HTN incidence was SBP ≥ 140 mmHg and/or DBP ≥ 90 mmHg or initiation of antihypertensive treatment, in line with JNC VII and international guidelines. On the other hand, regarding the prediction horizon, they ranged from 1 to 30 years, with windows of 4 to 10 years being more common.

### 3.3. Methodological Quality of Systematic Reviews

The methodological quality assessment of the six included systematic reviews revealed significant heterogeneity. We evaluated the risk of bias using the ROBIS tool, which assesses four key domains: study eligibility criteria, identification and selection of studies, data collection and study appraisal, and synthesis and findings (see Table 2).

Only Schjerven (2024) [4] demonstrated low risk of bias across all ROBIS domains, making it the most methodologically robust review in our analysis. The review featured a comprehensive search strategy, duplicated selection and extraction processes, appropriate use of the PROBAST tool for primary-study evaluation, and detailed exploration of heterogeneity. However, the review explicitly did not formally assess publication bias, which we considered when interpreting its pooled AUC estimates.

Chowdhury (2022) [2] showed a low risk of bias in three domains but received an “unclear” rating in the synthesis domain due to limitations in the exploration of heterogeneity, resulting in an overall “unclear” risk of bias. Despite having a registered protocol and using appropriate risk-of-bias assessment tools, the meta-analyses’ conclusions may be affected by insufficient investigation of the sources of heterogeneity.

The remaining four reviews—Echouffo-Tcheugui (2013) [22], Sun (2017) [21], Hamoen (2019) [3], and Silva (2022) [23]—were all assessed as having high overall risk of bias. Common limitations included incomplete reporting of search and selection methods, inconsistent assessment of risk of bias in primary studies, limited exploration of heterogeneity, and insufficient transparency in synthesis decisions; protocol registration was also inconsistently reported.

Methodological guidance for systematic reviews and meta-analyses of prediction-model performance recommends pooling performance estimates from validations of the same prediction model, or using methods that explicitly account for differences in model structure, population, outcome definition, and validation setting [24]. When AUCs from distinct models are pooled as if they were exchangeable replications, the resulting estimate describes an artificial average rather than any specific tool that can be implemented, recalibrated, or evaluated clinically. We therefore retained such meta-analyses as descriptive summaries of the published literature, but did not treat them as definitive evidence of the transportability of any individual model or model class.

The evaluation confirmed a pattern of methodological improvement in more recent reviews, with publications from 2022 onwards showing greater adherence to contemporary standards, including more consistent use of the PROBAST tool for evaluating primary studies. However, essential deficiencies in transparency and critical rigor persist across the body of evidence.

Overall, the available evidence on prognostic models for hypertension remains vulnerable to important methodological limitations, underscoring the need for registered protocols, fully duplicated processes, and rigorous bias assessments in future reviews. These findings highlight the importance of cautious interpretation of the conclusions from most of the included reviews, with greater confidence warranted for the results from Schjerven (2024) [4] due to its lower risk of bias.

An additional methodological limitation involved the mixing of cross-sectional diagnostic screening models and prognostic models in Sun [21] and Chowdhury [2]. Cross-sectional diagnostic/screening models aim to identify current or previously undiagnosed hypertension at the time of assessment, whereas prognostic models estimate future incident hypertension among individuals known to be normotensive at baseline. Combining their performance metrics reduces clinical interpretability because the target population, intended use, validation requirements, and decision context differ.

### 3.4. Overlap Between Systematic Reviews

The overlap analysis revealed a CCA of 17.8%, indicating a very high overlap among the included reviews. The primary studies included in Echouffo-Tcheugui et al. (2013) [22] were consistently included in Sun (2017) [21], Chowdhury et al. (2022) [2], and Schjerven et al. (2024) [4], while the latter two reviews show the highest degree of overlap with each other (68% of shared studies). Hamoen (2019) [3] and Silva (2022) [23] presented the least overlap with other reviews, reflecting their more specific focuses (childhood models and ML models, respectively).

The high overlap was managed at three levels. First, primary studies were counted once when summarizing the number of unique models to avoid inflating the apparent volume of evidence. Second, when multiple reviews covered the same primary studies, conclusions were weighted narratively toward the most recent and methodologically robust reviews rather than treating overlapping reviews as independent confirmation. Third, we conducted a restricted prognostic sensitivity synthesis excluding Sun [21] and Chowdhury [2], the two reviews that mixed diagnostic/screening and prognostic models. This sensitivity synthesis did not materially change the qualitative conclusions regarding limited external validation, sparse calibration reporting, and absence of clinical-impact evidence; however, it further reduced confidence in comparative AUC claims between traditional and machine-learning approaches. The results of the restricted prognostic sensitivity synthesis are summarized in Table 3.

### 3.5. Types of Prognostic Models Evaluated

From the six grouped reviews, we can summarize that the models are distributed into three broad categories:

Traditional statistical models: These are the majority, 97 models (60%), developed with logistic regression, Cox regression, or, to a lesser extent, Weibull. The Framingham Hypertension Risk Score, the Johns Hopkins/Precursors Study, the ARIC study model, the Korean KoGES, and the Tehran Lipid and Glucose Study were the most frequently evaluated. They are usually published as continuous equations, simple scores, or nomograms that facilitate risk estimation in clinical practice.

ML models: The most recent analyses document 48 models based on algorithms such as Support Vector Machines, Random Forests, neural networks, and XGBoost. These models incorporate a larger number of predictors and can capture non-linear interactions; however, external validation remains limited, and reproducibility is constrained by incomplete reporting of variable selection, hyperparameters, and model availability.

Genetic models (polygenic risk scores): 17 models (10%) were found, almost all synthesized in the review by Sun (2017) [21]. They integrate tens to hundreds of SNPs associated with hypertension; there is no consensus on the optimal panel of markers or the weighting method, and their clinical utility remains exploratory.

### 3.6. Predictors Included in the Models

Across the included reviews, core clinical predictors were consistently reported: age, baseline systolic blood pressure, body mass index or other anthropometric measures, sex, baseline diastolic blood pressure, family history of hypertension, smoking, glycaemic markers, lipid profile, and physical activity. Because predictor reporting was not harmonized across reviews, Figure 2 is restricted to the two model families for which Chowdhury et al. (2022) [2] reported explicit model-level frequencies: traditional regression-based models and machine-learning models. Accordingly, the figure provides a source-specific descriptive comparison and should not be interpreted as a pooled estimate across all 162 unique models identified in this umbrella review.

Traditional statistical models generally relied on compact predictor sets, whereas machine-learning models more frequently incorporated laboratory biomarkers, detailed lifestyle indices, socioeconomic variables, and higher-dimensional feature sets. Genetic/polygenic risk score models primarily used SNP-based scores, either alone or combined with conventional clinical predictors; however, no included review identified a consensus SNP panel or standardized weighting method. Figure 2 excludes genetic/polygenic risk-score models because harmonized model-level frequencies for convention-al predictors were unavailable.

### 3.7. Predictive Performance of the Models

In terms of discrimination, three reviews reported meta-analyses based on AUC. Schjerven (2024) [4] estimated pooled AUCs of 0.78 (95% CI 0.76–0.80) for traditional statistical models and 0.82 (95% CI 0.77–0.86) for ML algorithms. Chowdhury (2022) [2] obtained slightly lower values—0.75 (95% CI 0.73–0.77) for traditional models and 0.76 (95% CI 0.72–0.79) for ML—while Sun (2017) [21] reported a combined AUC of 0.77 (95% CI 0.74–0.79). However, these pooled AUCs should be interpreted as descriptive summaries rather than clinically stable performance estimates. The contributing reviews combined heterogeneous models, populations, prediction horizons, and validation settings, and all showed extreme heterogeneity (I^2^ ≥ 99%) with wide prediction intervals. Under these conditions, a single pooled AUC does not estimate the expected performance of a specific model in a specific target population.

Evaluation of publication bias or small-study effects was inconsistent. Sun (2017) [21] assessed funnel-plot asymmetry and Begg/Egger tests and reported no statistically significant publication bias, whereas Chowdhury (2022) [2] and Schjerven (2024) [4] explicitly did not assess publication bias. Consequently, optimistic discrimination due to small derivation studies or selective reporting cannot be excluded. Our interpretation therefore relies more heavily on the consistency of evidence regarding external validation, calibration, and clinical-impact evaluation than on pooled AUC values alone.

Calibration metrics were much less frequent than AUC: only 24 of the 162 unique models presented calibration slope or observed-expected curves. In the derivation cohorts, calibration was acceptable, but external validations tended to overestimate absolute risk, highlighting the need for recalibration before applying the models in clinical practice.

Regarding clinical utility, only seven models had been externally validated more than once, and even so, most had a single additional cohort. Only 35 models reported predictive values; the negative predictive value was generally high (>80%), while the positive predictive value ranged from 25% to 65%, reflecting the difficulty in accurately predicting who will develop hypertension in intermediate-risk populations.

### 3.8. External Validation

External validation was scarce. Only 21 of 94 models with extractable validation information had undergone at least one external validation (22%). Moreover, only seven of the 162 unique models identified across reviews had been externally validated more than once, indicating that robust evidence of transportability remains limited. Traditional statistical models were more likely to have been externally validated (27.3%) compared with ML models (8.7%) and PRS-based models (20.0%).

The Framingham model for HTN had the strongest validation record, with seventeen validations reported in diverse populations, although with variable performance (AUC between 0.537 and 0.840). Other models, including KoGES and several country- or cohort-specific scores, had substantially fewer validations.

Existing external validations frequently showed a decrease in predictive performance compared to derivation studies, with average reductions in AUC of 0.05–0.10, and often required recalibration for application in new populations. Particularly notable was the scarcity of validations in populations from Latin America, Africa, and parts of Asia, limiting the evidence on the transportability of these models to regions with increasing prevalence of HTN.

Regarding geographic and ethnic representation in external validations, the available evidence shows significant disparities. The Framingham model validations were predominantly conducted in high-income countries, with the majority performed in European populations (England, Germany, Denmark), followed by North American (USA, Canada) and Asian populations (China, Japan, Korea, Iran). Notably, only one validation study was identified in Latin America (Brazil) [25], and no external validations were found in African populations. This geographic distribution highlights the substantial underrepresentation of populations from regions with the highest burden of hypertension, limiting the generalizability of current evidence to global populations where these models could provide the greatest public health impact.

### 3.9. Clinical Impact and Utility

None of the six reviews identified studies—neither randomized trials nor observational evaluations—that analyzed the effect of using these models in healthcare practice (e.g., better blood pressure control, reduction in cardiovascular events, or more efficient use of resources). Schjerven (2024) [4] and Chowdhury (2022) [2] explicitly highlight this absence and point to it as a research priority.

### 3.10. Methodological Quality of Primary Studies

Three of the six included reviews formally assessed the quality of the primary studies. Schjerven (2024) [4] and Chowdhury (2022) [2] used the PROBAST tool, while Hamoen (2019) [3] applied the CHARMS framework to pediatric models.

In Schjerven (2024) [4], 53 studies were analyzed. Only four presented a low overall risk of bias. The Analysis domain concentrated the highest proportion of studies with high risk, followed by Participants; in contrast, Predictors and Outcomes showed lower levels of concern. The authors rated the overall methodological quality as “poor”, questioning the reliability of the published models. Chowdhury (2022) [2] reviewed 52 traditional regression studies; 19 studies were classified as low overall risk, while the rest presented applicability concerns because the models were developed in contexts poorly representative of usual clinical practice. Again, the most problematic domains were Analysis and Participant Selection, reflecting deficiencies in statistical handling and sample representativeness.

To synthesize both evaluations, a “traffic light” diagram was constructed (see Figure 3) that combines the results of PROBAST. The Analysis domain exhibits the highest proportion of high risk (80%), followed by Participants (53%). The Predictors and Outcomes domains perform better: around 58% and 70% of studies are classified as low risk. This pattern indicates that, even when the measurement of predictors and the definition of outcomes are usually adequate, deficiencies in statistical methods and sample selection undermine the validity of the models.

In the pediatric setting, Hamoen (2019) [3] detected with CHARMS a “recurrent lack of methodological information” and “suboptimal methods” in the 18 identified models. The absence of external validation and poor calibration reporting stand out, limiting the generalization of their results.

Grouping the findings, consistent patterns of methodological weakness are observed: (1) inadequate handling of missing data, with a predominance of list-wise deletion; (2) selection bias, with over-representation of Caucasian or Asian populations and scarce inclusion of vulnerable groups; (3) lack of blinding when measuring predictors and outcomes; (4) overfitting, especially in models with many predictors and few events; (5) limited or incompletely reported internal validation procedures, rarely based on bootstrapping or cross-validation; and (6) selective performance reporting, with emphasis on discrimination and frequent omission of calibration.

## 4. Discussion

### 4.1. Synthesis of Main Findings

This umbrella review identified a large number of prognostic models for incident hypertension, but limited evidence supporting their transportability and implementation in routine care. Across six systematic reviews, 162 models were described; however, only 21 of 94 models with extractable validation information had undergone at least one external validation, calibration was infrequently reported, and no clinical-impact studies were identified. These findings indicate that the main limitation of the field is not the absence of candidate models, but the insufficient evaluation of existing models in clinically relevant settings.

Although reported pooled AUC estimates suggested moderate discrimination for both traditional statistical and machine-learning models, these estimates should be interpreted cautiously. The contributing reviews combined heterogeneous models, populations, prediction horizons, and validation contexts, with extreme heterogeneity and wide prediction intervals. Therefore, pooled AUCs should be viewed as descriptive summaries of the published literature rather than as reliable estimates of the expected performance of any specific model in a target population.

Traditional statistical models remain the most frequently evaluated and externally validated, whereas machine-learning models show limited transparency and sparse external validation despite their apparent discriminative performance. Among individual models, the Framingham Hypertension Risk Score has the strongest validation record, but its evidence remains geographically restricted, with limited validation in Latin America and no identified validation in African populations.

### 4.2. Comparison with Previous Literature

Current hypertension guidelines conceptually recognize the value of risk stratification, but they do not recommend routine use of a specific model for incident hypertension. This position is consistent with the evidence identified in our review: discrimination has been reported often, but transportability, calibration, and clinical impact remain insufficiently evaluated.

The implementation gap contrasts with better established prediction tools in cardiovascular medicine, such as global cardiovascular risk scores, EuroSCORE II, CHA_2_DS_2_-VASc, and Wells-type clinical decision rules [26,27,28,29]. These tools achieved clinical adoption only after repeated validation, practical implementation formats, endorsement by professional societies, and linkage to clear decision thresholds. Hypertension prognostic models have not yet reached that level of evidence maturity.

Our findings also align with broader methodological literature showing that machine-learning models do not consistently outperform traditional regression models in clinical prediction and that poorly validated prediction models can remain at high risk of bias despite apparently acceptable discrimination [30,31]. In this context, model proliferation with limited validation evidence should not be mistaken for clinical readiness.

### 4.3. Critical Knowledge Gaps

The most important knowledge gap is the absence of clinical-impact studies. No included review identified randomized or observational evaluations testing whether use of a hypertension prediction model improves preventive decisions, blood-pressure outcomes, cardiovascular outcomes, or resource allocation. Without this step, model performance remains a statistical property rather than evidence of clinical utility [32,33].

The second major gap is limited external validation in diverse populations. Although 21 of 94 models with extractable information had at least one external validation, only seven of 162 unique models had been externally validated more than once. Evidence remains especially limited in Latin America, Africa, and several parts of Asia, where the burden of hypertension is high and where baseline risk, predictor distributions, health-system access, and preventive resources may differ substantially from derivation cohorts.

The third gap is inadequate calibration assessment and reporting. Discrimination alone cannot determine whether a model provides accurate absolute risk estimates [34,35]. This is particularly relevant for hypertension prevention because clinical decisions depend on whether predicted absolute risk is sufficiently reliable to justify lifestyle counseling intensity, follow-up frequency, or other preventive interventions.

Finally, the secondary evidence itself requires caution. Some prior reviews pooled performance estimates from heterogeneous models or mixed diagnostic/screening and prognostic models. Methodological guidance recommends separating the intended clinical use of models and pooling performance estimates only when the target model and validation context are sufficiently comparable [24]. Future reviews should therefore avoid treating heterogeneous AUC estimates as interchangeable evidence.

### 4.4. Implications for Practice and Research

Our findings support a pragmatic shift from repeated model derivation toward external validation, recalibration, implementation, and impact evaluation. Priority should be given to models with transparent equations or deployable code, conventional predictors available in primary care, repeated external validation, and adequate calibration after local updating [36,37,38].

This recommendation does not imply that every geographic region requires a de novo prediction model. Rather, each intended-use setting should externally validate and, when necessary, recalibrate existing models before routine implementation. New model derivation should be considered only when external validation demonstrates poor transportability despite recalibration or when essential locally relevant predictors are absent from existing models.

Risk prediction for incident hypertension should complement, not compete with, treatment of established hypertension. Its role is to support targeted prevention among normotensive individuals at elevated future risk, while diagnosis, treatment, and control of established hypertension remain parallel priorities.

For future research, the most efficient roadmap includes coordinated validation consortia in underrepresented populations, standardized reporting using TRIPOD or TRIPOD-AI, systematic calibration assessment, transparent model availability, and pragmatic clinical-impact studies in primary care. These steps are more likely to advance implementation than additional derivation studies in already well-represented settings.

### 4.5. Strengths and Limitations

This UR presents significant methodological strengths that increase the robustness of its conclusions. The design incorporated exhaustive search strategies in multiple databases and complementary sources, minimizing the risk of relevant omissions. The dual and independent assessment of risk of bias using ROBIS provides a structured evaluation of the methodological quality of the included reviews. The formal analysis of overlap using the CCA enabled the identification and mitigation of redundancy between reviews, adequately prioritizing those of higher quality and timeliness. Finally, incorporating comparability among traditional statistical, machine-learning, and genetic/polygenic approaches provides a comprehensive view of the current landscape.

However, the present UR has several limitations when interpreting its findings. First, we depended on the information reported in the included SRs, with potential loss of details from primary studies, particularly for methodological aspects that original reviewers did not prioritize. Second, the high degree of review overlap (CCA = 17.8%) indicates substantial redundancy; this was managed through narrative weighting and sensitivity synthesis, but it cannot be completely eliminated. Third, the methodological heterogeneity among the included SRs, with different inclusion criteria, search periods, and assessment tools, complicates integrated synthesis. Fourth, quantitative performance estimates should be interpreted cautiously given the extreme statistical heterogeneity (I^2^ > 99%), inconsistent assessment of publication bias, and the mixing of heterogeneous models in some original meta-analyses. Finally, although we attempted to minimize language bias through multilingual searches, we cannot completely rule out omission of reviews published in languages other than English or Spanish, particularly from underrepresented regions.

## 5. Conclusions

This umbrella review shows that the evidence base on prognostic models for incident hypertension has reached a point where development has clearly outpaced validation and clinical use. A total of 162 models have been described with apparent discriminative capacity, but there remain limited opportunities to use them in routine practice: only 22% have at least one external validation, calibration is reported in a minority of models, and no published clinical impact studies evaluating their effect on patient outcomes were identified. The methodological quality of the secondary literature itself shows substantial variability, with inappropriate mixing of diagnostic screening and prognostic models in some meta-analyses. Taken together, these findings indicate that the field is ready to move from deriving new models to validating and implementing the best existing ones—particularly in primary care settings where hypertension prevention is most relevant.

Based on our findings, we recommend a strategic shift in the approach to prognostic models for hypertension, with four fundamental actions: (1) avoid prioritizing additional de novo model derivation in already well-represented settings and redirect resources toward validating the most promising existing models; (2) prioritize external validation and recalibration in diverse populations, particularly in low- and middle-income countries where the prevention opportunity is greatest; (3) conduct pragmatic clinical-impact studies, including cluster-randomized trials, to quantify whether using these models in primary care improves hypertension prevention, blood-pressure outcomes, and resource allocation; and (4) integrate standardized reporting frameworks (TRIPOD-AI, PROBAST-AI) and calibration assessment into every new validation study. New model development should be reserved for settings where existing models show poor transportability after rigorous validation and recalibration, or where locally relevant predictors are absent from available models.

## Figures and Tables

**Figure 1:**
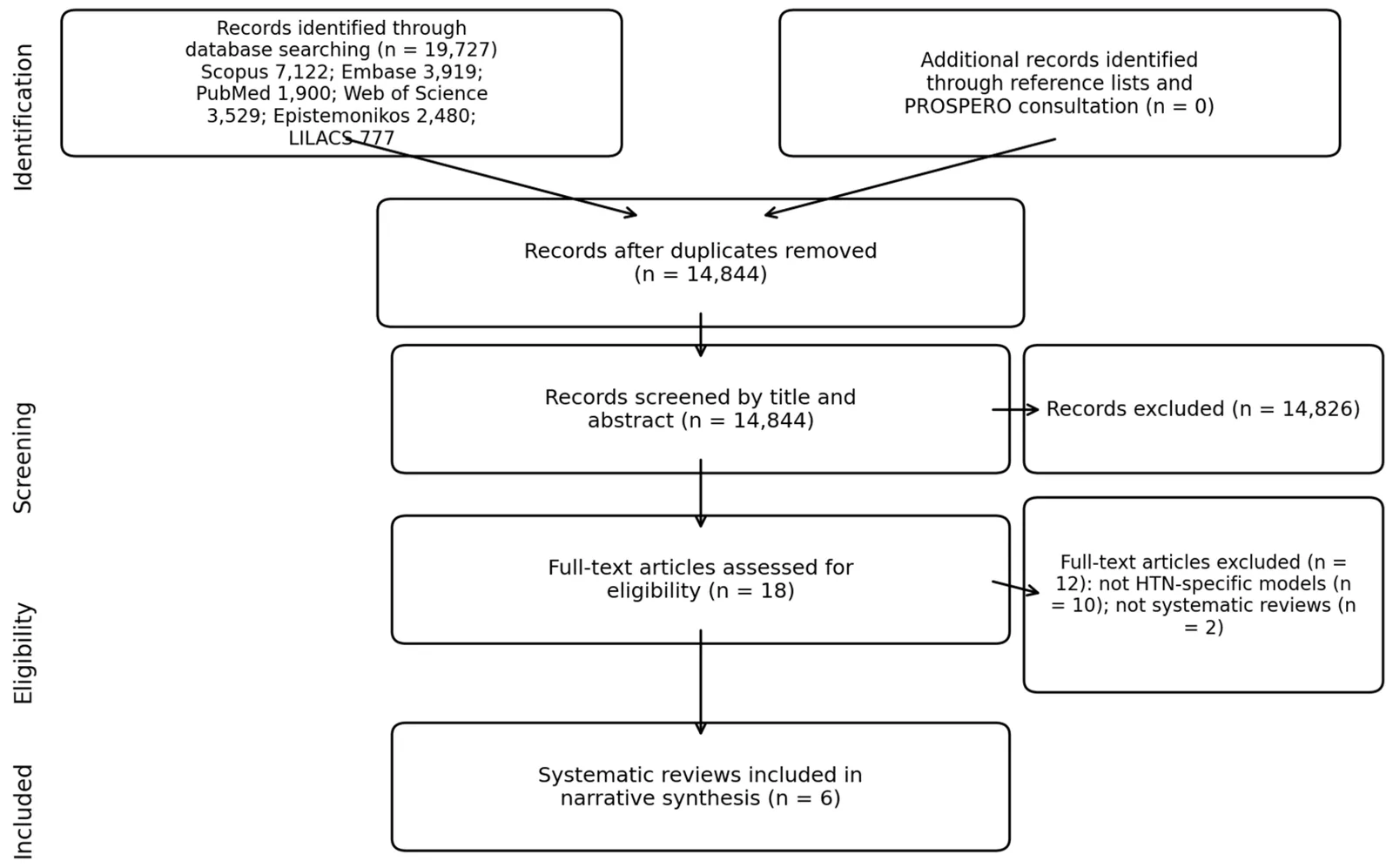
PRISMA flow diagram of study selection. The diagram reports records identified across six electronic databases, duplicate removal, title/abstract screening, full-text eligibility assessment, reasons for exclusion, and the six systematic reviews included in the narrative synthesis.

**Figure 2:**
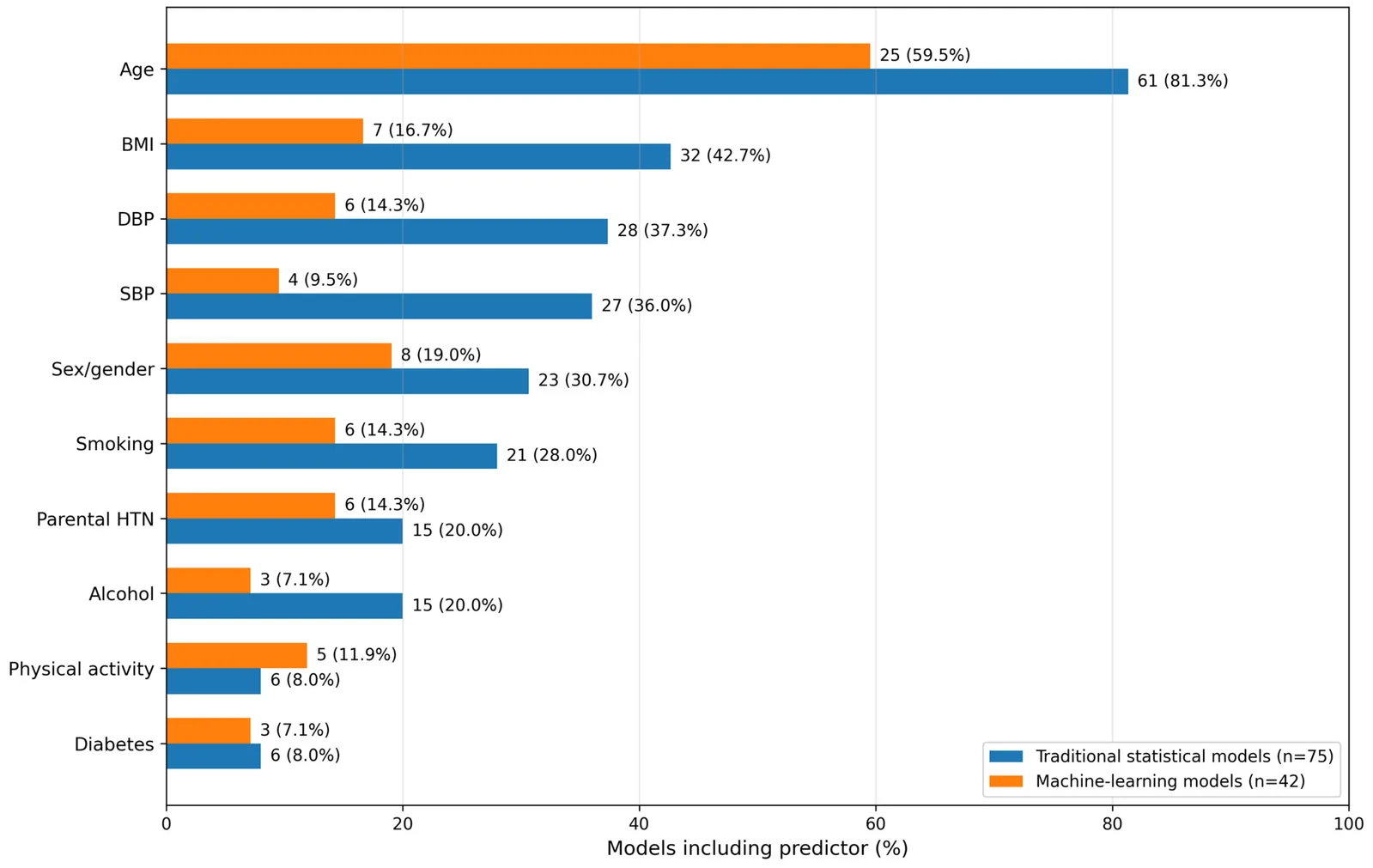
Frequency of conventional predictors in traditional regression-based and machine-learning hypertension prediction models. Data were adapted from Chowdhury et al. (2022) [2]. Bars represent the percentage of models within each model family that included each predictor, and data labels are presented as *n* (%); the denominators were 75 traditional regression-based models and 42 machine-learning models. Genetic/polygenic risk-score models were excluded from this figure because harmonized model-level frequencies for conventional predictors were unavailable. This source-specific figure should not be interpreted as a pooled estimate across the 162 unique models identified in this umbrella review. SBP = systolic blood pressure; DBP = diastolic blood pressure; BMI = body mass index; HTN = hypertension.

**Figure 3:**
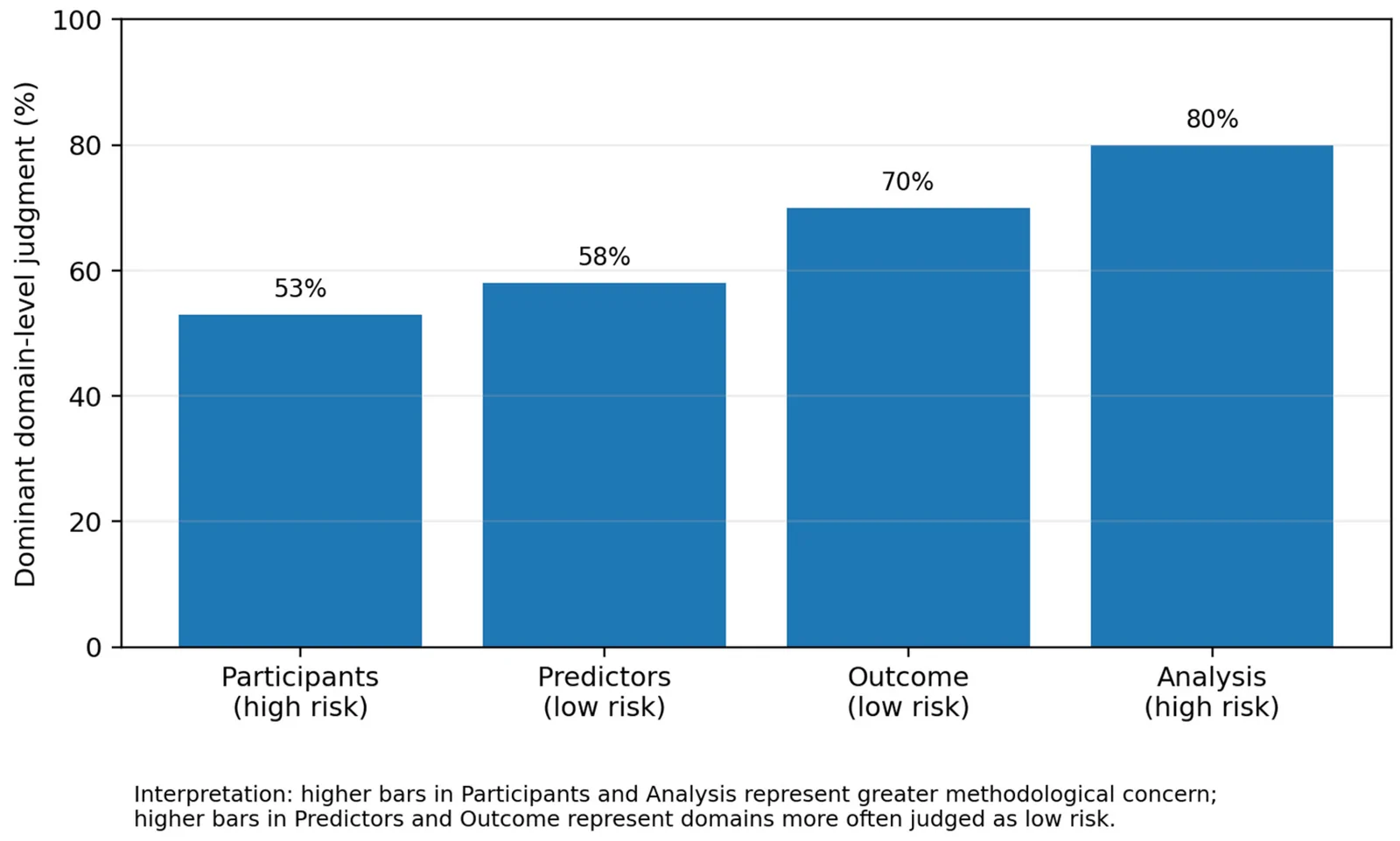
Risk-of-bias profile of primary prediction-model studies according to PROBAST domains. The figure summarizes the dominant domain-level concerns extracted from the included reviews that reported PROBAST assessments. The analysis domain concentrated the largest proportion of high-risk judgments, followed by participant selection, whereas predictor and outcome measurement were comparatively less problematic. PROBAST = Prediction model Risk Of Bias ASsessment Tool.

**Table 1:** Characteristics of included systematic reviews.

Review	Scope and Model Family	Search Coverage and Evidence Base	Population/HTN Definition	Quality Tool and Key Interpretive Note
Echouffo-Tcheugui 2013 [22]	Adult prognostic risk models; mainly traditional statistical models	Up to April 2013; 11 studies, 15 models	Adults; mostly North America/Europe; incident HTN generally SBP ≥ 140 mmHg and/or DBP ≥ 90 mmHg or treatment	No formal prediction-model risk-of-bias tool; retained as early descriptive evidence
Sun 2017 [21]	Updated review of hypertension risk-prediction models; traditional and genetic models; included some screening-oriented evidence	Up to September 2016; 26 studies, 48 models	Asia, North America, Europe; mostly adult cohorts; HTN generally SBP/DBP ≥ 140/90 mmHg or treatment	No formal risk-of-bias tool; publication bias assessed; excluded from primary prognostic-discrimination sensitivity synthesis
Hamoen 2019 [3]	Childhood/adolescent models predicting later hypertension	Up to March 2018; 6 studies, 18 models	Children/adolescents from Europe and North America; adult HTN or adolescent pre-HTN/HTN outcomes	CHARMS-based appraisal; pediatric scope, no external validation identified
Chowdhury 2022 [2]	Traditional regression and machine-learning models; included prognostic and some diagnostic/screening designs	Up to December 2020; 52 studies, 117 models; 32 in meta-analysis	General adult populations; mainly Asia, Europe, Americas; HTN usually SBP/DBP ≥ 140/90 mmHg or treatment	PROBAST; unclear ROBIS synthesis domain; excluded from primary prognostic-discrimination sensitivity synthesis
Silva 2022 [23]	Machine-learning models for hypertension prediction	January 2018–May 2021; 21 studies	Mostly Asia and North America; mixed clinical/data-science settings	No formal ROBIS-compatible tool reported; useful for ML transparency and implementation issues
Schjerven 2024 [4]	Adult prognostic risk models for incident hypertension; quantitative synthesis by model type	Up to March 2023; 53 studies; 47 in meta-analysis	15 countries; no Africa/Oceania representation; incident HTN generally SBP/DBP ≥ 140/90 mmHg or treatment	PROBAST; low ROBIS risk; prioritized for adult prognostic evidence but publication bias not formally assessed

SBP = Systolic Blood Pressure; DBP = Diastolic Blood Pressure; HTN = Hypertension; ML = Machine Learning; CHARMS = CHecklist for critical Appraisal and data extraction for systematic Reviews of prediction models; PROBAST = Prediction model Risk Of Bias Assessment Tool.

**Table 2:** Risk of bias assessment of included systematic reviews according to ROBIS.

Systematic Review (First Author and Year)	Study Eligibility Criteria	Identification and Selection of Studies	Data Collection and Appraisal	Synthesis and Findings	Overall Risk of Bias
Echouffo-Tcheugui 2013 [22]	Low	High	High	High	High
Sun 2017 [21]	Low	High	High	High	High
Hamoen 2019 [3]	Low	Low	High	High	High
Chowdhury 2022 [2]	Low	Low	Low	Unclear	Unclear
Silva 2022 [23]	Low	High	High	High	High
Schjerven 2024 [4]	Low	Low	Low	Low	Low

**Table 3:** Restricted prognostic sensitivity synthesis.

Sensitivity Set	Reviews Included for Primary Interpretation	Reviews Excluded/Deprioritized	Effect on Interpretation
Full descriptive map	All six reviews	None	Used to describe the breadth of the literature and model proliferation.
Restricted prognostic synthesis	Echouffo-Tcheugui 2013 [22]; Hamoen 2019 [3]; Silva 2022 [23]; Schjerven 2024 [4]	Sun 2017 [21] and Chowdhury 2022 [2] excluded from primary prognostic-discrimination inference because they mixed diagnostic/screening and prognostic models	Main conclusions unchanged: extensive model development, limited external validation, scarce calibration reporting, and no clinical-impact studies. Comparative AUC claims became less certain.
ROBIS-prioritized synthesis	Schjerven 2024 [4] prioritized for adult prognostic evidence; other reviews used descriptively according to scope and risk of bias	High-risk or highly overlapping reviews not treated as independent confirmation	Greater weight placed on validation, calibration, impact evidence, and heterogeneity rather than on pooled AUC alone.

## Data Availability

All data generated or analyzed during this umbrella review are included in this published article and its Supplementary Materials. The dataset consists of data extracted from the original systematic reviews, which are publicly available.

## Supplementary Materials

The following supporting information can be downloaded at: https://www.mdpi.com/article/10.3390/diagnostics16193069/s1, Supplementary Material S1: PRIOR 2022 checklist; Supplementary Material S2: Search strategy by database.
