# Supplementary material for: Prognostic Models for Incident Hypertension: Time to Move from Development to Implementation—An Umbrella Review
Source: Diagnostics (Basel). 2026 Sep 22;16(19):3069. doi: 10.3390/diagnostics16193069 (PMC13664178; doi:10.3390/diagnostics16193069)
Supplement: Supplementary file 1 [file diagnostics-16-03069-s001.zip › Supplementary Material S1.pdf]

**Supplementary Material S1.** PRIOR 2022 Checklist.

| Section / Topic | Item # | Checklist item | Location in manuscript / Notes |
|---|---|---|---|
| **Title** | **Title** | **Title** | **Title** |
| Title | **1** | Identify the report as an overview of reviews. | Title page explicitly states "An Umbrella Review" in the main title, which is an accepted synonym for overview of reviews in the PRIOR terminology. |
| **Abstract** | **Abstract** | **Abstract** | **Abstract** |
| Abstract | **2** | Provide a comprehensive and accurate summary of the purpose, methods, and results of the overview of reviews. | Structured Abstract (first page): Introduction, Objective, Methods, Results, Conclusions, and Keywords. |
| **Introduction** | **Introduction** | **Introduction** | **Introduction** |
| Rationale | **3** | Describe the rationale for conducting the overview of reviews in the context of existing knowledge. | Introduction, paragraphs 1–4 (global burden of HTN, proliferation of prognostic models, heterogeneity among existing SRs, gap in primary-care implementation). |
| Objectives | **4** | Provide an explicit statement of the objective(s) or question(s) addressed by the overview of reviews. | Introduction, final paragraph ("The present umbrella review aims to synthesize the available evidence…"). Abstract also contains an explicit objective statement. |
| **Methods** | **Methods** | **Methods** | **Methods** |
| Eligibility criteria | **5a** | Specify the inclusion and exclusion criteria for the overview of reviews. If supplemental primary studies were included, this should be stated, with a rationale. | Section 2.2 Eligibility criteria (inclusion: SRs of multivariable models predicting incident HTN in normotensive adults with at least one externally validated model; six explicit exclusion criteria). No supplemental primary studies were included; the unit of analysis was the systematic review. |
| | **5b** | Specify the definition of "systematic review" as used in the inclusion criteria for the overview of reviews. | Reported in Section 2.2. The manuscript now defines a systematic review as a review with explicit eligibility criteria, a reproducible search strategy, |

| | | | |
|---|---|---|---|
| | | | structured study selection, and systematic synthesis; narrative reviews/editorials without explicit systematic methods were excluded. |
| Information sources | **6** | Specify all databases, registers, websites, organisations, reference lists, and other sources searched or consulted to identify systematic reviews and supplemental primary studies (if included). Specify the date when each source was last searched or consulted. | The inconsistency has been corrected. Both Abstract and Methods now state six databases, with searches from January 2000 to March 2026. MEDLINE/PubMed, Embase, Scopus, Web of Science, LILACS, and Epistemonikos are all listed consistently. |
| Search strategy | **7** | Present the full search strategies for all databases, registers and websites, such that they could be reproduced. Describe any search filters and limits applied. | Section on Information sources and search strategy (tripartite conceptual structure: HTN, prediction/prognostic models, SR methodological filter). Full strategies, Boolean operators, truncations, search fields, and limiters documented in Supplementary Material 2. |
| Selection process | **8a** | Describe the methods used to decide whether a systematic review or supplemental primary study (if included) met the inclusion criteria of the overview of reviews. | Section Study selection: deduplication and screening in Rayyan QCRI with reviewer blinding; dual independent title/abstract and full-text screening by VJVP and JBC; standardized eligibility form; disagreements resolved by direct discussion between the two reviewers. |
| | **8b** | Describe how overlap in the populations, interventions, comparators, and/or outcomes of systematic reviews was identified and managed during study selection. | Reported. Reviews with overlapping PICO were retained, and primary-study overlap was quantified post-selection by CCA. The manuscript now explains that overlap was managed by counting unique models once, weighting conclusions toward more recent/methodologically robust reviews, and |

| | | | |
|---|---|---|---|
| | | | conducting a restricted prognostic sensitivity synthesis. |
| Data collection process | **9a** | Describe the methods used to collect data from reports. | Section Data extraction: standardized REDCap template with six modules (bibliographic, SR methodology, model attributes, performance, validation, conclusions); piloted with three SRs; dual independent extraction by VJVP and JBC with biostatistician consultation for complex metrics; conflict resolution record documented. |
| | **9b** | If applicable, describe the methods used to identify and manage primary study overlap at the level of the comparison and outcome during data collection. For each outcome, specify the method used to illustrate and/or quantify the degree of primary study overlap across systematic reviews. | Clarified. CCA was calculated globally across the included systematic reviews because the overview did not perform separate outcome-level meta-analyses. The interpretation and sensitivity synthesis are now described in the main manuscript. |
| | **9c** | If applicable, specify the methods used to manage discrepant data across systematic reviews during data collection. | The manuscript now clarifies that discrepant or overlapping evidence was handled narratively: the most recent and methodologically robust review was prioritized, no new quantitative pooling was performed, and reviews mixing diagnostic and prognostic models were excluded from primary prognostic-discrimination inference. |
| Data items | **10** | List and define all variables and outcomes for which data were sought. Describe any assumptions made and/or measures taken to identify and clarify missing or unclear information. | Clarified in the revised manuscript. Missing or unclear information from included reviews was recorded as not reported; no assumptions were made to impute unreported model-performance, calibration, or validation data. |

| | | | |
|---|---|---|---|
| Risk of bias assessment | **11a** | Describe the methods used to assess risk of bias or methodological quality of the included systematic reviews. | The revised manuscript consistently reports ROBIS as the tool used to assess risk of bias in the included systematic reviews; the Strengths and Limitations section now matches the Methods and Results. |
| | **11b** | Describe the methods used to collect data on (from the systematic reviews) and/or assess the risk of bias of the primary studies included in the systematic reviews. Provide a justification for instances where flawed, incomplete, or missing assessments are identified but not reassessed. | Section Evaluation of methodological quality and risk of bias: PROBAST (and PROBAST-AI where applicable) assessments were extracted from the included SRs in a "review of evaluations" approach, aggregating the proportion of primary studies at low/moderate/high RoB per domain. No reassessment of primary studies that lacked PROBAST was performed; the justification for this (inherent dependence on reporting by included SRs) is implicit in the Limitations section but could be made explicit. |
| | **11c** | Describe the methods used to assess the risk of bias of supplemental primary studies (if included). | Not applicable. No supplemental primary studies were included. |
| Synthesis methods | **12a** | Describe the methods used to summarise or synthesise results and provide a rationale for the choice(s). | Section Results synthesis: structured narrative synthesis with categorization by model type (traditional statistical, ML, genetic); AUCs and prediction intervals extracted from the original reviews without new quantitative pooling, with the rationale that repooling could introduce additional biases given primary-study overlap. Sensitivity analyses excluding redundant reviews are also mentioned. |
| | **12b** | Describe any methods used to explore possible causes of heterogeneity among results. | Clarified. Overview-level heterogeneity exploration was narrative and prespecified by model family, |

| | | | |
|---|---|---|---|
| | | | review risk of bias, overlap, and intended use (prognostic vs diagnostic/screening). No new pooled meta-analysis was performed because original syntheses had unit-of-analysis limitations and high overlap. |
| | **12c** | Describe any sensitivity analyses conducted to assess the robustness of the synthesised results. | Section Overlap between SRs explicitly mentions sensitivity analyses excluding reviews with higher redundancy and prioritizing the most recent and methodologically robust reviews (Schjerven 2024, Chowdhury 2022). |
| Reporting bias assessment | **13** | Describe the methods used to collect data on (from the systematic reviews) and/or assess the risk of bias due to missing results in a summary or synthesis (arising from reporting biases at the levels of the systematic reviews, primary studies, and supplemental primary studies, if included). | Updated. The revised manuscript now states that Sun 2017 assessed funnel-plot asymmetry and Begg/Egger tests and reported no statistically significant publication bias, whereas Chowdhury 2022 and Schjerven 2024 did not formally assess publication bias. The implications for optimistic discrimination estimates are discussed. |
| Certainty assessment | **14** | Describe the methods used to collect data on (from the systematic reviews) and/or assess certainty (or confidence) in the body of evidence for an outcome. | No formal certainty-of-evidence framework was applied. The revised manuscript provides a structured narrative confidence assessment by outcome: pooled AUC estimates are treated as descriptive and lower-confidence because of heterogeneity and model non-exchangeability, whereas the absence of clinical-impact studies and scarcity of external validation are treated as consistent high-salience findings. |
| **Results** | **Results** | **Results** | **Results** |
| Systematic review and supplemental primary study selection | **15a** | Describe the results of the search and selection process, including the number of records screened, assessed for eligibility, and included in the overview of | Section Study selection: 19,727 records identified, 14,844 after de-duplication, 18 full-text assessed, 6 included. Figure 1 presents the PRISMA flow diagram. |

| | | | |
|---|---|---|---|
| | | reviews, ideally with a flow diagram. | |
| | **15b** | Provide a list of studies that might appear to meet the inclusion criteria, but were excluded, with the main reason for exclusion. | Section 3.1 provides aggregate full-text exclusion reasons: 10 records were excluded because they were not specific to hypertension prediction models and 2 because they were not systematic reviews. |
| Characteristics of systematic reviews and supplemental primary studies | **16** | Cite each included systematic review and supplemental primary study (if included) and present its characteristics. | Section Characteristics of included SRs and Table 1: six reviews cited (EchouffoTcheugui 2013, Sun 2017, Hamoen 2019, Chowdhury 2022, Silva 2022, Schjerven 2024) with objective, search coverage, number of studies/models, populations, HTN definition, and quality tool used. |
| Primary study overlap | **17** | Describe the extent of primary study overlap across the included systematic reviews. | Section 3.4 reports CCA = 17.8%, describes pairwise overlap narratively, and explains how overlap was managed by counting unique models once, prioritizing more robust reviews, and conducting a restricted prognostic sensitivity synthesis. |
| Risk of bias in systematic reviews, primary studies, and supplemental primary studies | **18a** | Present assessments of risk of bias or methodological quality for each included systematic review. | Section Methodological quality of the SRs and Table 2: ROBIS domain-level and overall judgments for each of the six reviews (EchouffoTcheugui and Sun high RoB; Hamoen and Silva high; Chowdhury unclear; Schjerven low). |
| | **18b** | Present assessments (collected from systematic reviews or assessed anew) of the risk of bias of the primary studies included in the systematic reviews. | Section Methodological quality of primary studies and Figure 3 (traffic-light diagram): aggregated PROBAST domains — Analysis 80% high RoB, Participants 53% high RoB, Predictors ~58% low, Outcomes ~70% low. CHARMS findings for Hamoen 2019 (pediatric) also reported. |

| | | | |
|---|---|---|---|
| | **18c** | Present assessments of the risk of bias of supplemental primary studies (if included). | Not applicable. No supplemental primary studies were included. |
| Summary or synthesis of results | **19a** | For all outcomes, summarise the evidence from the systematic reviews and supplemental primary studies (if included). If meta-analyses were done, present for each the summary estimate and its precision and measures of statistical heterogeneity. If comparing groups, describe the direction of the effect. | external validation in 21/94 models with extractable validation information (22%); only 7/162 unique models had been externally validated more than once (approximately 4%); no clinical-impact studies identified. |
| | **19b** | If meta-analyses were done, present results of all investigations of possible causes of heterogeneity. | Partially reported. Original-review heterogeneity results ($I^2 \geq 99\%$) are narrated. Explicit investigation of causes (subgroups, meta-regression) was not performed at the overview level, consistent with the narrative synthesis approach. The manuscript attributes heterogeneity to unit-of-analysis errors in the original meta-analyses (mixing different models; mixing prediction horizons). |
| | **19c** | If meta-analyses were done, present results of all sensitivity analyses conducted to assess the robustness of synthesised results. | Section Overlap between SRs: sensitivity analysis prioritizing Schjerven 2024 and Chowdhury 2022 after excluding the most redundant reviews is described; the narrative conclusions remained stable. |
| Reporting biases | **20** | Present assessments (collected from systematic reviews and/or assessed anew) of the risk of bias due to missing primary studies, analyses, or results in a summary or synthesis (arising from reporting biases at the levels of the systematic reviews, primary | Section Predictive performance now provides a structured reporting-bias statement: Sun 2017 assessed publication bias and found no statistically significant asymmetry, whereas Chowdhury 2022 and Schjerven 2024 did not formally assess publication |

| | | | |
|---|---|---|---|
| | | studies, and supplemental primary studies, if included) for each summary or synthesis assessed. | bias. The overview did not perform new funnel/Egger analyses because pooled AUCs were not based on exchangeable units. |
| Certainty of evidence | **21** | Present assessments (collected or assessed anew) of certainty (or confidence) in the body of evidence for each outcome. | A formal GRADE summary table was not added. The revised Discussion and Limitations provide narrative certainty judgments for the main outcomes: discrimination estimates are interpreted cautiously, whereas the validation, calibration, and clinical-impact gaps are presented as the most robust conclusions. |
| **Discussion** | **Discussion** | **Discussion** | **Discussion** |
| Discussion | **22a** | Summarise the main findings, including any discrepancies in findings across the included systematic reviews and supplemental primary studies (if included). | Section Synthesis of main findings: development of prognostic models has clearly outpaced external validation and clinical implementation; extreme heterogeneity across models; only 22% externally validated; 15% reported calibration; no published clinical-impact studies were identified. Under 'Comparison with previous literature' and 'Critical knowledge gaps', findings are positioned against other successful cardiovascular risk scores (Framingham, SCORE2, Wells) to illustrate the validation and implementation gap. Limitations discussed in section 'Strengths and Limitations' (inherited from included reviews; reliance on published data; evolving field). |
| | **22b** | Provide a general interpretation of the results in the context of other evidence. | Section Comparison with previous literature: contrast with successful cardiovascular prediction models (Framingham global risk, EuroSCORE, $CHA_2DS_2$-VASc, Wells), convergence with Christodoulou et al. on ML, and |

| | | | |
|---|---|---|---|
| | | | with Wynants et al. on COVID-19 prediction models. |
| | **22c** | Discuss any limitations of the evidence from systematic reviews, their primary studies, and supplemental primary studies (if included) included in the overview of reviews. Discuss any limitations of the overview of reviews methods used. | Corrected. Strengths and Limitations now refers to ROBIS only and discusses limitations related to overlap, heterogeneity, publication-bias assessment, and reliance on reported data from included reviews. |
| | **22d** | Discuss implications for practice, policy, and future research (both systematic reviews and primary research). Consider the relevance of the findings to the end users of the overview of reviews, eg, healthcare providers, policymakers, patients, among others. | Section Implications for practice and research and Conclusions and Recommendations: four-phase roadmap (identification, coordinated external validation, statistical recalibration, clinical-impact evaluation); specific guidance for primary care and low- and middle-income settings; recommendations for scientific societies, funders, journals, and healthcare systems. |
| **Other information** | **Other information** | **Other information** | **Other information** |
| Registration and protocol | **23a** | Provide registration information for the overview of reviews, including register name and registration number, or state that the overview of reviews was not registered. | The revised manuscript states that the umbrella review was not prospectively registered and that methods were guided by PRIOR and documented in the manuscript and supplementary materials. |
| | **23b** | Indicate where the overview of reviews protocol can be accessed, or state that a protocol was not prepared. | No separate public protocol was prepared. The revised manuscript states this transparently and provides the methodological details in the main text and supplementary materials. |
| | **23c** | Describe and explain any amendments to information provided at registration or in the protocol. Indicate the stage of the overview of reviews at which amendments were made. | Not applicable if no protocol exists. Otherwise not reported. |

| | | | |
|---|---|---|---|
| Support | **24** | Describe sources of financial or non-financial support for the overview of reviews, and the role of the funders or sponsors in the overview of reviews. | Funding declaration: article processing charges to be covered by Vicerrectorado de Investigación, UNTRM; funder had no role in design, data collection, analysis, decision to publish, or manuscript preparation. |
| Competing interests | **25** | Declare any competing interests of the overview of reviews' authors. | Conflict of interest declaration: "The authors declare that they have no conflict of interest." |
| Author information | **26a** | Provide contact information for the corresponding author. | Title page: Víctor Juan Vera-Ponce, victor.vera@untrm.edu.pe, ORCID 0000-0003-4075-9049. |
| | **26b** | Describe the contributions of individual authors and identify the guarantor of the overview of reviews. | Updated. The revised Author Contributions section now states that V.J.V.-P. accepts responsibility as guarantor for the integrity of the review and the decision to submit the manuscript. |
| Availability of data and other materials | **27** | Report which of the following are available, where they can be found, and under which conditions they may be accessed: template data collection forms; data collected from included systematic reviews and supplemental primary studies; analytic code; any other materials used in the overview of reviews. | The Data Availability Statement now states that extracted data are included in the article and supplementary materials and that the study-review overlap matrix and sensitivity-synthesis decisions are available from the corresponding author upon reasonable request. |

PRIOR: Preferred Reporting Items for Overviews of Reviews. Source: Gates M, Gates A, Pieper D, et al. Reporting guideline for overviews of reviews of healthcare interventions: development of the PRIOR statement. BMJ 2022;378:e070849.
