# Supplementary material for: Prognostic Models for Incident Hypertension: Time to Move from Development to Implementation—An Umbrella Review
Source: Diagnostics (Basel). 2026 Sep 22;16(19):3069. doi: 10.3390/diagnostics16193069 (PMC13664178; doi:10.3390/diagnostics16193069)
Supplement: Supplementary file 1 [file diagnostics-16-03069-s001.zip › Supplementary Material S2.pdf]

Supplementary Material S2. Search strategy by database.

All searches were run from January 2000 to March 2026 with no language restrictions. Six electronic databases were searched: PubMed/MEDLINE, Scopus, Web of Science, Embase, Epistemonikos, and LILACS/BVS.

PubMed

Table S2.1. Search strategy in PubMed.

Strategy	Search terms
#1	("Hypertension"[Mesh] OR hypertens*[tiab] OR "high blood pressure"[tiab] OR "arterial hypertension"[tiab])
#2	(model*[tiab] OR score*[tiab] OR nomogram*[tiab] OR algorithm*[tiab] OR equation*[tiab] OR "machine learning"[tiab] OR "deep learning"[tiab] OR "artificial intelligence"[tiab])
#3	(predict*[tiab] OR risk[tiab] OR prognos*[tiab])
#4	(systematic[sb] OR "systematic review"[ti] OR "systematic reviews"[ti] OR meta-analysis[pt] OR meta-analys*[ti] OR "umbrella review"[ti] OR "overview of reviews"[ti])
#5	#1 AND #2 AND #3 AND #4

Scopus

Table S2.2. Search strategy in Scopus.

Strategy	Search terms
#1	TITLE-ABS-KEY (hypertens* OR "high blood pressure" OR "arterial hypertension")
#2	TITLE-ABS-KEY (model* OR score* OR nomogram* OR algorithm* OR equation* OR "machine learning" OR "deep learning" OR "artificial intelligence")
#3	TITLE-ABS-KEY (predict* OR risk OR prognos*)
#4	TITLE-ABS-KEY ("systematic review" OR "systematic reviews" OR meta-analysis OR meta-analyses OR "meta analysis" OR "umbrella review" OR "overview of reviews")
#5	#1 AND #2 AND #3 AND #4

Web of Science

Table S2.3. Search strategy in Web of Science.

Strategy	Search terms
#1	TS=(hypertens* OR "high blood pressure" OR "arterial hypertension")
#2	TS=(model* OR score* OR nomogram* OR algorithm* OR equation* OR "machine learning" OR "deep learning" OR "artificial intelligence")
#3	TS=(predict* OR risk OR prognos*)

#4	TS=("systematic review" OR "systematic reviews" OR meta-analysis OR meta-analyses OR "meta analysis" OR "umbrella review" OR "overview of reviews")
#5	#1 AND #2 AND #3 AND #4

Embase

Table S2.4. Search strategy in Embase.

Strategy	Search terms
#1	(hypertension:ti,ab,kw OR 'high blood pressure':ti,ab,kw OR 'arterial hypertension':ti,ab,kw)
#2	(model:ti,ab,kw OR score:ti,ab,kw OR nomogram:ti,ab,kw OR algorithm:ti,ab,kw OR equation:ti,ab,kw OR 'machine learning':ti,ab,kw OR 'deep learning':ti,ab,kw OR 'artificial intelligence':ti,ab,kw)
#3	(predict*:ti,ab,kw OR risk:ti,ab,kw OR prognos*:ti,ab,kw)
#4	('systematic review':ti,ab,kw OR 'systematic reviews':ti,ab,kw OR 'meta analyses':ti,ab,kw OR 'meta analysis':ti,ab,kw OR 'umbrella review':ti,ab,kw OR 'overview of reviews':ti,ab,kw)
#5	#1 AND #2 AND #3 AND #4

Epistemonikos

Table S2.5. Search strategy in Epistemonikos.

Strategy	Search terms
#1	(title:(hypertension OR "high blood pressure" OR "arterial hypertension") OR abstract:(hypertension OR "high blood pressure" OR "arterial hypertension"))
#2	(title:(model OR score OR nomogram OR algorithm OR equation OR "machine learning" OR "deep learning" OR "artificial intelligence") OR abstract:(model OR score OR nomogram OR algorithm OR equation OR "machine learning" OR "deep learning" OR "artificial intelligence"))
#3	(title:(predict* OR risk OR prognos*) OR abstract:(predict* OR risk OR prognos*))
#4	(title:("systematic review" OR "systematic reviews" OR meta-analysis OR meta-analyses OR "meta analysis" OR "umbrella review" OR "overview of reviews") OR abstract:("systematic review" OR "systematic reviews" OR meta-analysis OR meta-analyses OR "meta analysis" OR "umbrella review" OR "overview of reviews"))
#5	#1 AND #2 AND #3 AND #4

LILACS/BVS

Table S2.6. Search strategy in LILACS/BVS.

Strategy	Search terms
#1	tw:(hypertens* OR "high blood pressure" OR "elevated blood pressure")

#2	tw:(model* OR score* OR nomogram* OR algorithm* OR equation* OR "machine learning" OR "deep learning" OR "artificial intelligence")
#3	tw:(predict* OR prognos* OR risk)
#4	tw:("systematic review" OR "systematic reviews" OR meta-analysis OR meta-analyses OR "meta analysis")
#5	#1 AND #2 AND #3 AND #4
